# Causal associations between plasma miRNAS and allergic rhinitis: A two-sample Mendelian randomization and functional enrichment study

**DOI:** 10.1016/j.bjorl.2026.101913

**Published:** 2026-09-22

**Authors:** Huaien Zhou, Jianjun Zou, Shidong Chu

**Affiliations:** Hangzhou Red Cross Hospital (Zhejiang Hospital of Integrated Traditional Chinese and Western Medicine), Department of Otolaryngology, Hangzhou, Zhejiang, China

**Keywords:** Allergic rhinitis, microRNAs, Mendelian randomization, Bayesian colocalization, Enrichment analyses

## Abstract

**Objective:**

Allergic rhinitis is a prevalent inflammatory disorder driven by complex immune dysregulation. While circulating miRNAs are known regulators of gene expression, their specific causal roles in the pathogenesis of allergic rhinitis remain largely undefined.

**Methods:**

We performed a comprehensive two-sample Mendelian randomization analysis to investigate the causal effect of plasma miRNAs on allergic rhinitis. Genetic instruments were obtained from large-scale miRNA eQTL datasets. Causal estimates were primarily evaluated using allergic rhinitis summary statistics from the GWAS catalog dataset and robustly validated in the independent FinnGen cohort (15,569 cases and 474,650 controls). Sensitivity analyses, including Bayesian colocalization and reverse Mendelian randomization, were conducted to ensure validity. Downstream mechanisms were explored via enrichment analyses of predicted targets.

**Results:**

We identified significant causal associations for multiple miRNAs. Notably, miR-130a-3p was consistently validated as a risk factor for allergic rhinitis across cohorts, whereas miR-1908-5p, miR-146a-3p, and miR-6891-3p exhibited protective effects. Colocalization analysis offered suggestive evidence of shared genetic variants for miR-148a-3p and the miR-181 family, with no evidence of reverse causality. Functional enrichment analysis revealed that target genes of these miRNAs are significantly involved in Wnt signaling, MAPK signaling, focal adhesion, and neurogenesis, implicating them in epithelial barrier regulation and neurogenic inflammation.

**Conclusions:**

This study provides genetic support for potential causal associations between circulating miRNAs and allergic rhinitis risk, prioritizing miR-130a-3p and miR-1908-5p as candidate regulators that warrant further functional validation.

**Level of Evidence:**

Level 5. Mendelian Randomized (MR) studies are second only to randomized controlled trials in terms of the level of evidence.

## Highlights

• Mendelian randomization reveals miR-130a-3p as allergic rhinitis risk factor.
• Protective causal effects of miR-1908-5p and miR-146a-3p in allergic rhinitis.
• Colocalization supports shared variants for miR-181 family.
• Targets of causal miRNAs enriched in Wnt, MAPK, and focal adhesion pathways.
• Plasma miRNAs offer allergic rhinitis biomarker and therapeutic potential.

## Introduction

Allergic rhinitis (AR) is a prevalent IgE-mediated inflammatory disorder affecting 10%–40% of the global population, imposing a substantial burden on quality of life and healthcare systems.^1^ While Genome-Wide Association Studies (GWASs) have identified multiple susceptibility loci, these explain only a modest proportion of AR heritability, prompting interest in post-transcriptional regulators like non-coding RNAs that integrate genetic and environmental risks.^2^

MicroRNAs (miRNAs) are small non-coding RNAs that fine-tune immune responses by repressing target mRNAs. Dysregulated miRNA expression is implicated in allergic pathogenesis, modulating T-helper cell polarization and epithelial responses.^2^^,^^3^ Mechanistic studies have highlighted specific miRNAs (e.g., miR-21, miR-146a) as regulators of airway inflammation,^4^^,^^5^ and genetic polymorphisms in miRNA genes have been associated with AR susceptibility.^6^

Beyond local tissues, circulating miRNAs are stable in peripheral blood and serve as potential non-invasive biomarkers. Although profiling studies indicate that specific plasma or serum miRNAs correlate with AR diagnosis, severity, and treatment response,^7^, ^8^, ^9^ these observational findings are often derived from small cross-sectional cohorts. Consequently, it remains unclear whether altered circulating miRNAs are causal drivers of AR, downstream consequences, or confounded epiphenomena.

Mendelian randomization (MR) strengthens causal inference by using genetic variants as instrumental variables (IVs),^10^ a strategy recently applied to prioritize inflammatory targets in AR.^11^ Leveraging the heritability of circulating miRNAs via cis-expression Quantitative Trait Loci (cis-miR-eQTLs) allows for the interrogation of their causal roles in complex diseases.^12^ However, this approach has not yet been applied to AR. Therefore, we performed a two-sample MR analysis using *cis*-acting genetic variants to systematically evaluate the potential causal effects of circulating miRNAs on AR risk.

## Methods

### Data sources for exposures and outcomes

The genetic instruments for circulating miRNAs were obtained from two independent large-scale GWAS to establish a robust two-stage analysis framework. The discovery phase utilized cis-Quantitative Trait Loci (cis-QTLs) from the Nikpay et al. Dataset (n = 710),^12^ while the validation phase incorporated trans-QTLs from the Nikpay et al. study and cis-QTLs from the Framingham Heart Study (FHS, n = 5239),^13^ all of which were standardized to the hg38 genome assembly. For the outcome phenotype, summary statistics for AR were acquired from two distinct and independent cohorts to ensure the reproducibility of findings: the FinnGen consortium (Release 12), comprising 15,569 cases and 474,650 controls, and the EBI GWAS Catalog (study accession GCST90476021), which included a larger cohort of 92,310 cases and 311,377 controls. The workflow diagram was illustrated in Fig. 1.

**Fig. 1:**
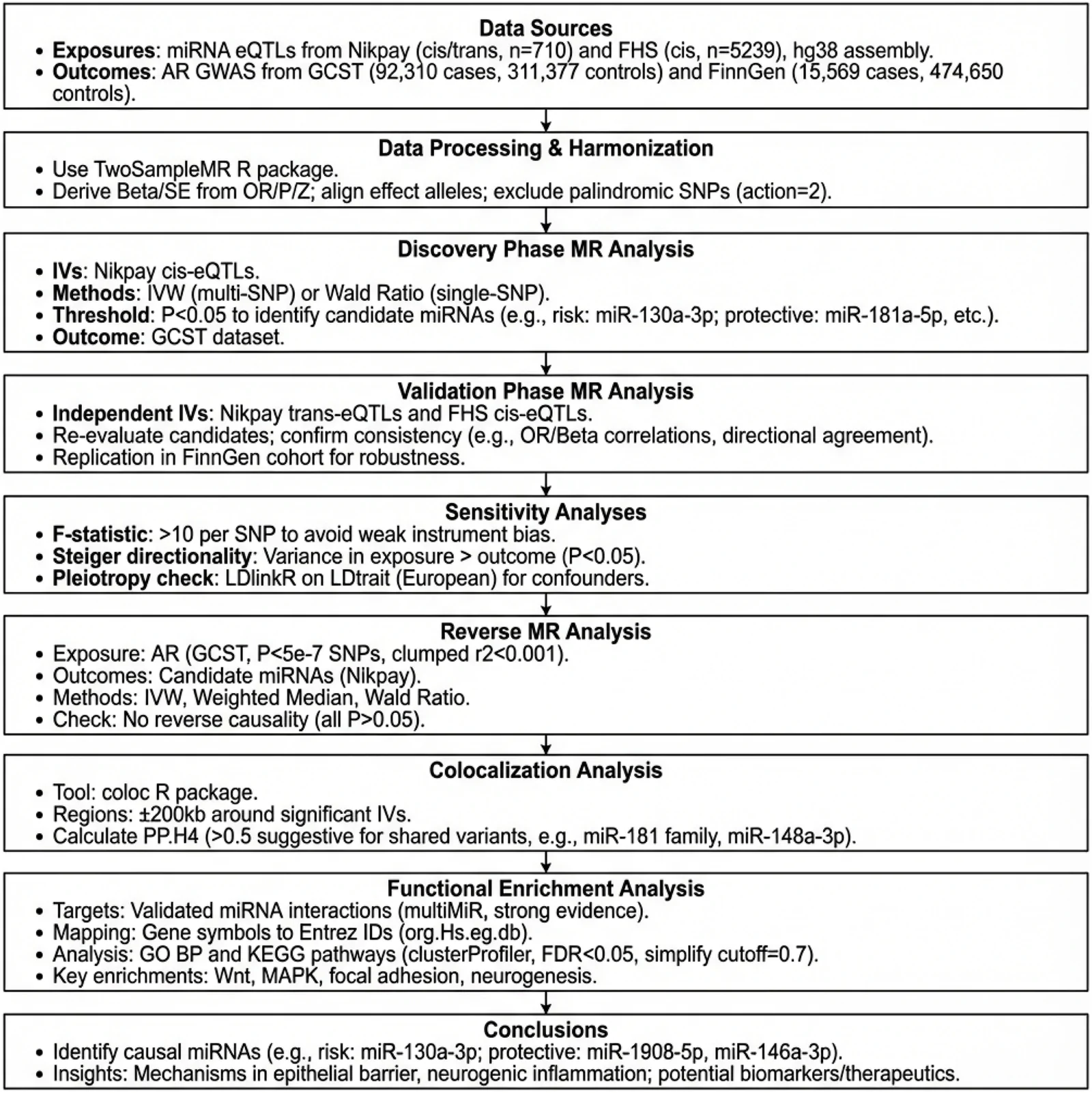
The workflow diagram of the study.

### Data processing and harmonization

Prior to the MR analysis, rigorous data pre-processing and harmonization were conducted using the TwoSampleMR R package. For the GCST outcome dataset, where standard errors were not directly provided, effect sizes (Beta) were derived by log-transforming the Odds Ratios (OR), and standard errors were subsequently estimated based on the reported p-values and Z-scores to ensure data completeness. The exposure and outcome datasets were harmonized by aligning effect alleles, and palindromic Single Nucleotide Polymorphisms (SNPs) with ambiguous allele frequencies were excluded (action = 2) to prevent strand alignment errors, ensuring that only valid genetic instruments were retained for analysis.

### Mendelian randomization analysis design

A two-stage MR design was implemented to identify and validate potential causal associations between miRNAs and allergic rhinitis. In the discovery phase, cis-QTLs from the Nikpay dataset were analyzed against both AR outcome datasets using the inverse variance weighted (IVW) method for instruments with multiple SNPs and the Wald Ratio method for single-SNP instruments, with a significance threshold set at p < 0.05. miRNAs identified as significant in the discovery phase were subsequently advanced to the validation phase, where their causal effects were re-evaluated using independent genetic instruments (Nikpay trans-QTLs and FHS cis-QTLs) to confirm the robustness of the associations and minimize the risk of false-positive results.

### Sensitivity analysis and pleiotropy assessment

To verify the validity of the causal inferences, comprehensive sensitivity analyses and pleiotropy checks were performed. The strength of the genetic instruments was evaluated by calculating the F-statistic for each SNP, and the Steiger directionality test was applied to confirm that the genetic instruments explained significantly more variance in the exposure (miRNAs) than in the outcome (AR). Additionally, to assess potential horizontal pleiotropy, the LDlinkR package was utilized to query the LDtrait database (European population), systematically screening significant instrumental variables for known associations with potential confounders.

### Reverse Mendelian randomization

To investigate potential reverse causality, we performed a reverse MR analysis treating AR as the exposure and the candidate miRNAs identified in the primary analysis as outcomes. Summary statistics for AR were obtained from the GCST90476021 dataset, while outcome data for miRNAs were retrieved from the Nikpay et al. study. Genetic instruments for AR were selected based on a genome-wide significance threshold of p < 5e-7, with effect sizes derived by log-transforming odds ratios, and were clumped for independence (*r*^2^ < 0.001, 10,000 kb window) using the 1000 Genomes European reference panel. Following data harmonization, causal estimates were generated using the IVW method, supplemented by Weighted Median and Wald Ratio tests, to determine whether genetic liability to AR influences circulating miRNA levels.

### Colocalization analysis

To distinguish true causal associations from spurious correlations driven by linkage disequilibrium (LD), we performed Bayesian colocalization analysis using the coloc R package. We focused on the genomic regions centered on the significant *cis*-acting instrumental variables (±200 kb) identified in the MR analysis. Summary statistics for allergic rhinitis were obtained from the GCST dataset, with effect sizes (Beta) and variances derived from reported odds ratios and confidence intervals, while corresponding miRNA statistics were retrieved from the Nikpay et al. dataset and converted to the hg38 assembly using rtracklayer to ensure coordinate alignment. For each miRNA-AR pair, we calculated the Posterior Probability of Hypothesis-4 (PP.H4), which represents the likelihood that both traits share a single causal variant, considering a PP.H4 > 0.8 as strong evidence of colocalization to reinforce the validity of the genetic instruments.

### Functional annotation and target enrichment analysis

To elucidate the biological mechanisms underlying the causal associations identified by Mendelian randomization, we performed a comprehensive functional enrichment analysis of the downstream targets of the significant miRNAs. Experimentally validated miRNA-target interactions were retrieved using the multiMiR R package, strictly filtering for targets supported by strong experimental evidence (e.g., reporter assays, Western blots) to ensure biological reliability. The resulting gene symbols were mapped to Entrez IDs using the org.Hs.eg.db database, and a comparative enrichment analysis was conducted using the compareCluster function within the clusterProfiler package to simultaneously evaluate gene ontology (GO) biological processes and Kyoto Encyclopedia of Genes and Genomes (KEGG) pathways across multiple miRNAs. Statistical significance was determined using the Benjamini-Hochberg method for false discovery rate (FDR) correction (adjusted p < 0.05), with redundant GO terms consolidated using the simplify function (cutoff = 0.7) to enhance interpretability, allowing for the visualization of shared and distinct biological pathways implicated in allergic rhinitis pathogenesis.

## Results

### Causal associations of genetically predicted miRNAs with allergic rhinitis risk

#### Selection of instrumental variables

In the discovery phase using the Nikpay cis-eQTL dataset, the F-statistics for all selected SNPs ranged from 22.08–271.50 (Supplementary Table S1). The Steiger directionality test confirmed that the variance explained by the IVs in the exposure was greater than in the outcome for all identified significant associations (Steiger_Pval < 0.05, Supplementary Table S1), supporting the correct causal direction. As illustrated in Fig. 2A, we identified 11 miRNAs that showed statistically significant causal associations with AR (p < 0.05), including two risky factors (miR-130a-3p and miR-148a-3p) and 9 protective miRNAs (miR-185-5p, miR-30b-5p, miR-181a-3p, miR-1908-5p, miR-181a-5p, miR-181b-5p, miR-451a, miR-30d-5p and miR-941). In the Nikpay Trans validation, seven miRNAs (miR-181a-5p, miR-30d-5p, miR-181b-5p, miR-1908-5p, miR-130a-3p, miR-451a and miR-148a-3p) replicated the significant associations observed in the discovery phase. The scatter plots demonstrated a strong positive correlation between the effect sizes derived from different datasets (Fig. 2B). Specifically, the correlation coefficient (RR) between the Discovery phase and the Nikpay Trans validation was 0.71 (p = 0.0042), and the correlation with the FHS Cis validation was 0.81 (p = 0.19, noting the smaller number of overlapping miRNAs). Crucially, as shown in the combined consistency analysis (Fig. 2B, bottom panel), 100% of the validated miRNAs (18/18 comparisons) showed consistent effect directions (all points located in the first or third quadrants). The overall correlation across all validation comparisons was robust (*R* = 0.74, p = 4.9e-4), reinforcing the validity of these miRNAs as putative causal biomarkers for allergic rhinitis.

**Fig. 2:**
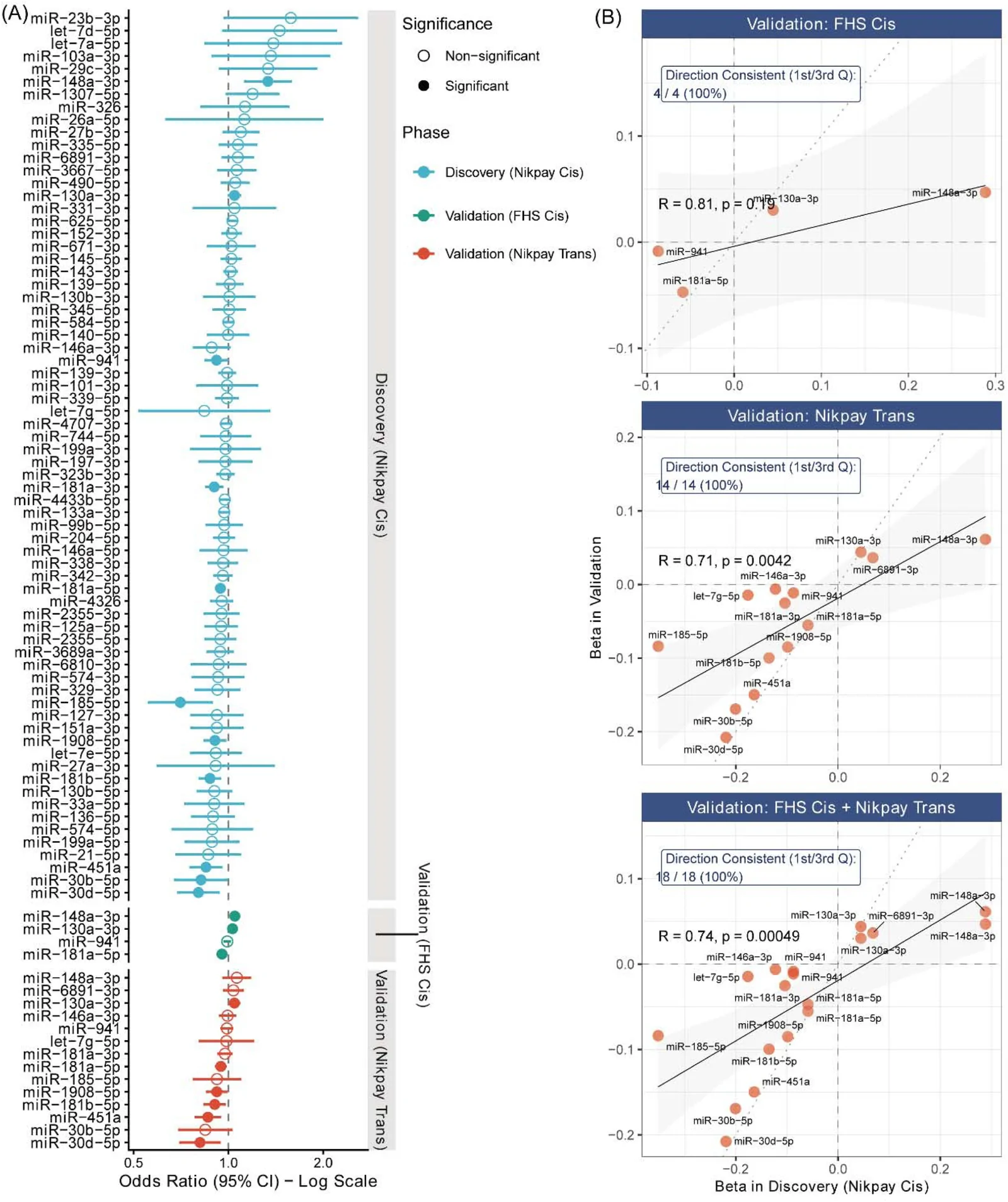
Causal associations of genetically predicted miRNAs with allergic rhinitis risk in the discovery and initial validation phases. (A) Forest plot displaying the Mendelian Randomization (MR) estimates for significant miRNAs identified in the discovery phase (using Nikpay Cis-eQTLs) and replicated in the validation phases (using Nikpay Trans-eQTLs and FHS Cis-eQTLs). The outcome dataset is the GCST90476021 GWAS summary statistics. Points represent the Odds Ratios (OR), and error bars indicate the 95% Confidence Intervals (95% CI). (B) Consistency analysis of causal effect sizes (Beta coefficients) between the discovery and validation phases. The x-axis represents the effect size in the discovery phase (Nikpay Cis), while the y-axis represents the effect size in the validation phases (FHS Cis and Nikpay Trans). The regression line (solid black) with the grey shaded area represents the 95% CI. The correlation coefficient (RR) and PP-value indicate the strength of the linear relationship. Points located in the first (upper-right) and third (lower-left) quadrants indicate directional consistency between datasets.

### Colocalization analysis between causal miRNAs and allergic rhinitis

Bayesian colocalization analysis revealed suggestive evidence of colocalization for three miRNAs. Specifically, miR-181b-5p and miR-181a-5p, which map to the same genomic locus on chromosome 1 (lead SNP rs12125200), exhibited posterior probabilities of a shared causal variant (PP.H4) of 0.586 and 0.581, respectively. Similarly, miR-148a-3p on chromosome 7 (lead SNP rs9639523) showed a PP.H4 of 0.564. Although these values fall below the strict threshold of 0.8, a PP.H4 greater than 0.5 is generally considered supportive of a shared genetic mechanism, thereby providing supportive, although not definitive, evidence for a shared genetic basis underlying the MR findings for these specific miRNAs. In contrast, miR-451a, miR-30d-5p, and miR-130a-3p showed limited evidence of colocalization, with PP.H4 values of 0.180, 0.095, and 0.021, respectively. However, the posterior probabilities for distinct independent signals (PP.H3) remained low (<0.025) for all examined associations. This suggests that the lack of strong colocalization support for these miRNAs might be attributed to limited statistical power in the outcome GWAS dataset or complex LD structures in these regions, rather than definitive evidence of distinct causal variants.

### Assessment of reverse causality

We utilized 9 independent genetic variants associated with AR (GCST90476021) as instrumental variables to assess their causal effects on the seven prioritized miRNAs. The results, detailed in Table S3, demonstrated no statistically significant evidence of a reverse causal effect of AR on any of the target miRNAs. Using the primary IVW method, the p-values for all associations were greater than 0.05. Consistent null results were obtained using the Weighted Median method for all tested miRNAs (all p > 0.05, Table S3).

### Validation of causal associations in the independent FinnGen cohort

As summarized in Table S4 and visualized in Fig. 3A, five miRNAs demonstrated significant causal associations with AR in the FinnGen cohort, including three protective miRNAs (miR-1908-5p, miR-146a-3p and miR-6891-3p) and two risky factors (let-7g-5p and miR-130a-3p). All significant IVs passed the weak instrument test (F-statistic > 10) and Steiger directionality test (p < 0.05). For the validation using FHS Cis instruments, the correlation of Beta coefficients between the two cohorts was strong (*R* = 0.91, p = 0.089), with 100% (4/4) of the miRNAs showing consistent effect directions (Fig. 3B, top panel). When combining all validation strategies (FHS Cis and Nikpay Trans), the overall directional consistency remained high at 83.3% (15/18), although the linear correlation was moderate (*R* = 0.23) due to heterogeneity in effect sizes between the distinct cohorts (Fig. 3B, bottom panel). Nevertheless, the preservation of the risk effect for miR-130a-3p and the protective trends for other key miRNAs across independent datasets provides compelling evidence for their biological relevance in the pathogenesis of allergic rhinitis.

**Fig. 3:**
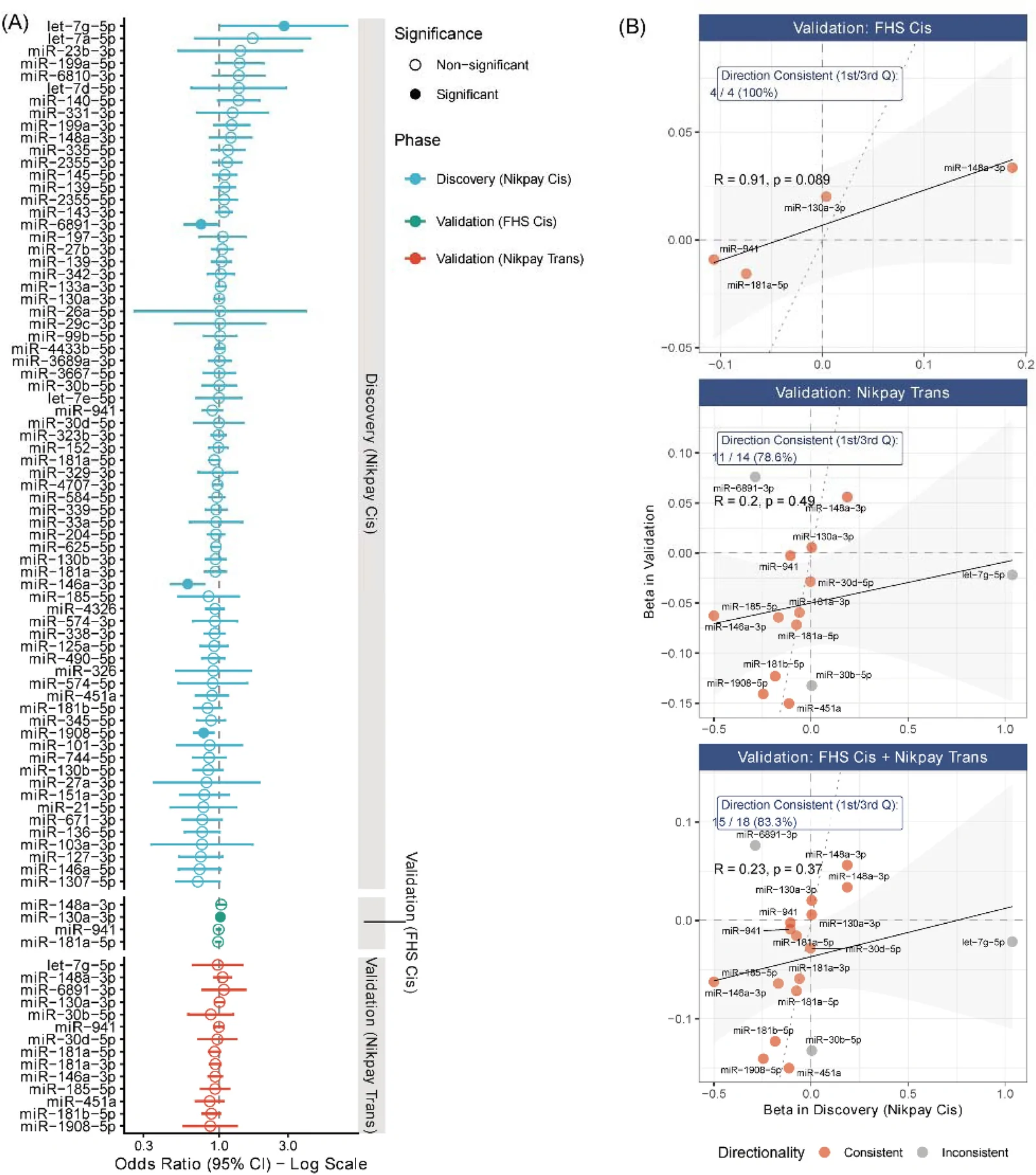
Independent validation of causal miRNAs in the FinnGen cohort. (A) Forest plot showing the replication results of causal miRNAs using the FinnGen R12 dataset as the outcome. Points represent ORs, and error bars denote 95% CIs. (B) Scatter plots illustrating the directional consistency of causal estimates between the primary discovery phase (GCST outcome) and the independent replication phase (FinnGen outcome). The consistency rate (percentage of points in the 1st/3rd quadrants) and Pearson correlation coefficients (RR) are provided to demonstrate the robustness of the identified causal signals across different populations.

### Functional annotation and enrichment analysis of prioritized miRNA targets

A total of 5212 unique target genes were identified, comprising 5123 targets for miR-130a-3p and 135 targets for miR-1908-5p (Supplementary Table S5). As visualized in the clustering tree diagram (Fig. 4), the targets were significantly enriched in diverse biological functions, which clustered into several key functional modules, including Signal Transduction and Phosphorylation, Developmental and Wnt Signaling, Neurogenesis and Synaptic Function, and Protein Catabolism and Response to Stimulus. The results of KEGG enrichment analysis highlighted several pathways closely related to immune regulation, cellular adhesion, and inflammatory responses (Fig. 5). Collectively, these functional enrichment results suggest that miR-130a-3p and miR-1908-5p may influence allergic rhinitis susceptibility by modulating genes involved in epithelial barrier function (focal adhesion), neurogenic mechanisms (synapse organization), and key inflammatory signaling cascades (MAPK, Wnt, TGF-beta).

**Fig. 4:**
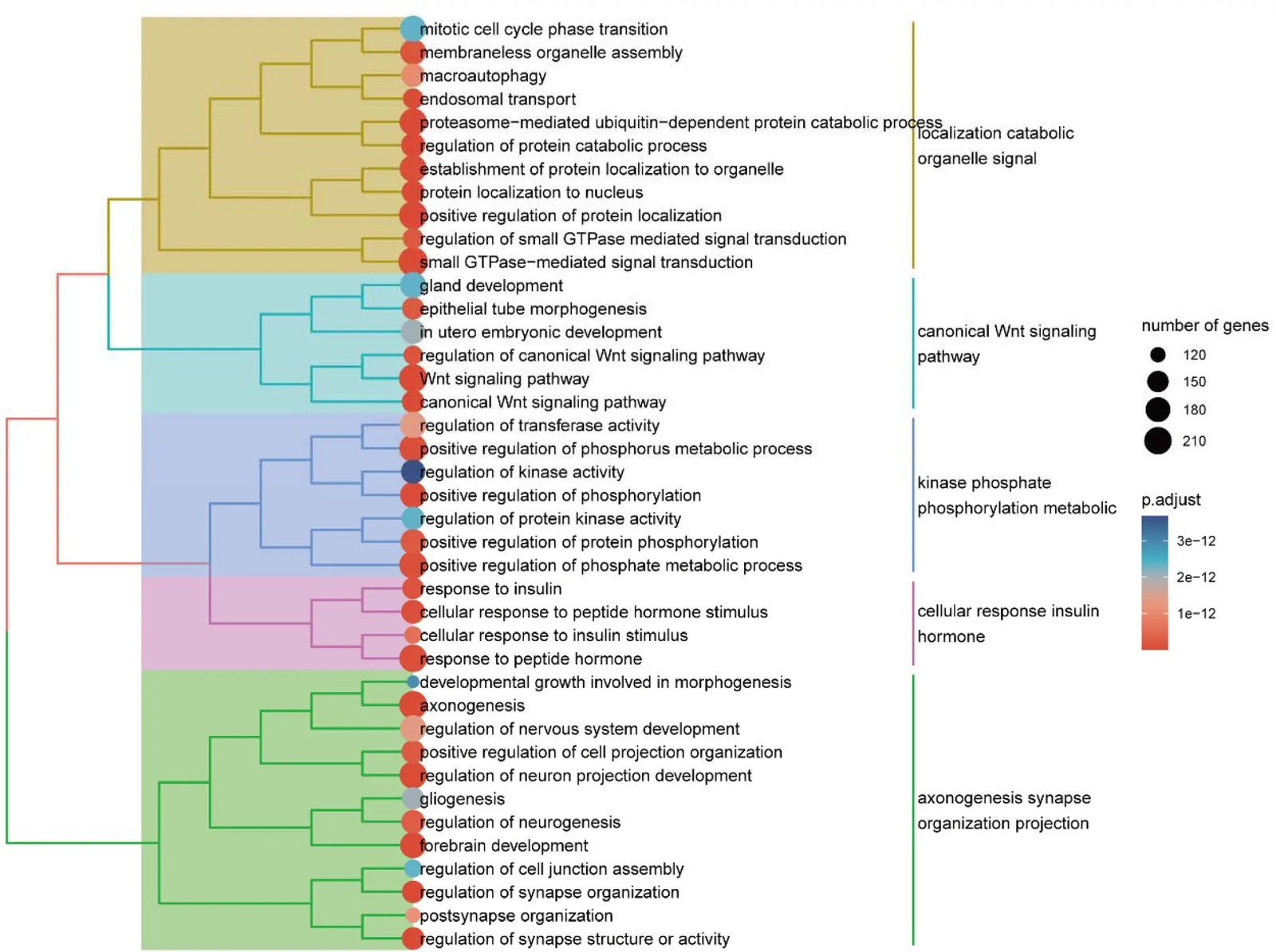
Gene ontology (GO) enrichment analysis of downstream targets for prioritized miRNAs. Hierarchical clustering tree summarizing the biological processes (BP) significantly enriched by the predicted target genes of the two robustly validated miRNAs (miR-130a-3p and miR-1908−5p). Branches of the tree represent the clustering of functionally related terms based on gene overlap, colored by functional modules (e.g., signal transduction, Wnt signaling, neurogenesis). The size of the dots corresponds to the number of genes enriched in each term, and the color gradient represents the statistical significance (adjusted PP-value), with redder colors indicating higher significance.

**Fig. 5:**
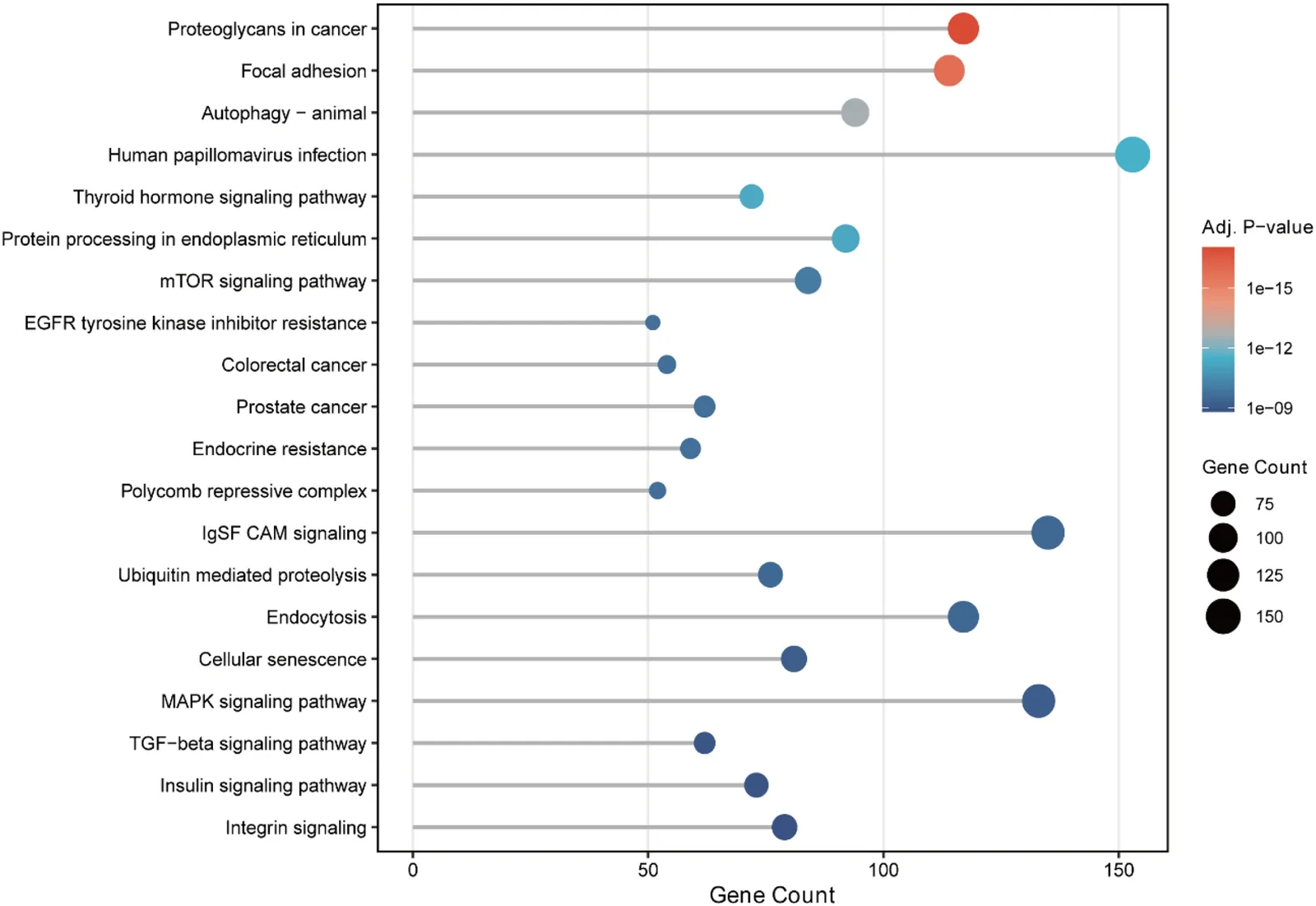
KEGG pathway enrichment analysis of prioritized miRNA targets. Lollipop chart displaying the top significantly enriched signaling pathways identified by KEGG analysis for the target genes of miR-130a-3p and miR-1908-5p. The y-axis lists the pathway names, and the x-axis represents the number of genes enriched in each pathway (“Gene Count”). The size of the bubbles is proportional to the gene count, while the color gradient indicates the significance level (adjusted PP-value), ranging from blue (lower significance) to red (higher significance).

## Discussion

Using a two‑sample MR framework based on large plasma miRNA eQTL resources and two independent AR GWASs, we identified a panel of circulating miRNAs with putative causal effects on AR risk. Notably, miR‑181a‑5p, miR‑181b‑5p, miR‑451a, miR‑30d‑5p, and miR‑1908‑5p consistently emerged as protective, whereas miR‑130a‑3p and miR‑148a‑3p were robustly associated with increased AR risk. These findings were replicated across multiple cohorts (GCST, FinnGen) and instrument sets (Nikpay et al., FHS), with sensitivity analyses supporting a causal direction from miRNA to AR.

The strongest protective signals were observed for the miR‑181 family (miR‑181a/b‑5p), a finding highly consistent with their known immunoregulatory functions. miR‑181a acts as a “rheostat” for T‑Cell Receptor (TCR) signaling; its expression modulates activation thresholds during thymic selection, shaping the naïve T‑cell repertoire and self‑reactivity.^14^^,^^15^ Beyond T‑cell development, miR‑181a/b exerts anti‑inflammatory effects in peripheral tissues by suppressing NF‑κB activation and restraining eosinophilic inflammation.^16^^,^^17^ In airway disease models, miR‑181b‑5p targets SPP1/osteopontin to modulate type‑2 cytokines, while its loss correlates with eosinophilia.^16^^,^^18^ Crucially, miR‑181a‑5p overexpression has been shown to directly alleviate symptoms in AR models by correcting Treg/Th17 imbalance and delaying the AR-asthma transition.^19^ Our MR results therefore support a model where life‑long upregulation of miR‑181 family expression protects against atopic sensitization by fine‑tuning T‑cell sensitivity and limiting airway inflammation.

We also found consistent protective effects for miR‑146a‑3p. miR‑146a is a canonical negative regulator of the TLR/NF‑κB pathway. In AR models and patients, it is often downregulated, while its restoration inhibits inflammation and promotes regulatory T‑cell (Treg) differentiation.^20^^,^^21^ Although some observational studies report elevated serum exosomal miR‑146a‑3p in AR patients as a marker of severity,^9^ our MR findings suggest its causal role is protective. This aligns with mechanistic data showing that miR‑146a‑3p promotes anti‑inflammatory M2 macrophage polarization and suppresses inflammasome activation.^22^^,^^23^ Thus, genetically higher baseline miR‑146a‑3p likely counteracts type‑2 inflammation.

Similarly, miR‑451a showed a protective association. While known to be elevated in acute anaphylaxis,^24^ functional studies indicate that miR‑451a restrains Th2 differentiation and pro‑asthmatic macrophage activation.^25^^,^^26^ The apparent contrast with observational data suggests that while acute release may mark severe reactions, higher genetic baseline levels confer protection. Other protective miRNAs identified offer novel mechanistic insights: miR‑30d‑5p regulates macrophage polarization^27^^,^^28^; miR‑185‑5p exerts anti‑inflammatory effects in airway smooth muscle;^29^^,^^30^ and miR‑1908‑5p, located in the FADS1 locus, has been linked to favorable cardiometabolic profiles, suggesting it may protect against AR by shaping an anti‑inflammatory immunometabolic milieu.^31^ Additionally, the protective effect of miR‑6891‑3p, encoded within the HLA‑B locus, implies a role in mucosal IgA homeostasis.^32^

Regarding risk factors, miR‑130a‑3p and miR‑148a‑3p were robustly supported. miR‑130a‑3p has previously been identified by MR as a causal risk factor for rheumatoid arthritis and Chronic Rhinosinusitis (CRS), suggesting it acts as a shared upstream driver of chronic mucosal and systemic inflammation, potentially via TGF‑β and mTOR signaling.^33^, ^34^, ^35^ miR‑148a is a critical regulator of B‑cell tolerance; its overexpression impairs tolerance checkpoints and promotes autoimmunity.^36^ In the context of AR, genetically higher miR‑148a‑3p may facilitate allergen‑reactive B‑cell survival and IgE class switching. We also observed a risk association for let‑7g‑5p; however, given conflicting literature regarding the let‑7 family’s role in asthma,^37^, ^38^, ^39^ this finding warrants cautious interpretation.

Our study advances the field by moving beyond observational correlations, which are prone to reverse causation.^9^ The miRNAs implicated here map to key nodes in allergic pathogenesis: (i) T‑cell selection and tolerance (miR‑181, miR‑185)^40^; (ii) Innate immune regulation (miR‑146a, miR‑30d, miR‑451a)^20^; (iii) B‑cell responses (miR‑148a, miR‑6891)^36^; and (iv) Immunometabolism (miR‑1908).^41^ The convergence of our findings with prior MR studies on miR‑130a‑3p and miR‑1908‑5p in other complex traits underscores their broad biological relevance.^12^^,^^35^

Several limitations exist. First, as eQTLs were derived from blood, we cannot fully resolve tissue‑specific mechanisms in the nasal mucosa, and circulating miRNAs may not completely reflect the local epithelial and immune processes directly involved in allergic rhinitis pathogenesis. Therefore, the identified miRNAs should be interpreted primarily as genetically proxied systemic biomarkers or regulators rather than definitive mediators of local nasal disease biology. Second, although we performed colocalization analyses, the evidence was only suggestive for several loci and did not consistently reach a stringent PP.H4 threshold of 0.8, which weakens confidence that the same causal variant drives both miRNA abundance and allergic rhinitis risk in all cases. Third, despite robust sensitivity analyses, including the use of cis-instruments where possible, F-statistic assessment, Steiger directionality testing, and LDtrait screening, horizontal pleiotropy remains a possibility, particularly in immune-related genomic regions. Fourth, because this study relied on summary-level data, we were unable to perform individual-level stratified analyses by age, sex, disease severity, sensitization subtype, comorbid asthma, or treatment status, and could not explore non-linear or mediation effects. Fifth, some nominal associations (e.g., miR‑30b‑5p, miR‑941) should be viewed as exploratory.^42^^,^^43^ Sixth, the focus on European ancestry and the absence of an Asian institutional or external validation cohort limit generalizability to non-European populations. Finally, MR estimates represent life‑long genetic exposure and may not capture dynamic changes during acute allergen exposure, and our functional annotation analyses were bioinformatic rather than experimental. Thus, although our findings are useful for prioritizing candidate miRNAs, direct mechanistic studies in relevant tissues and experimental systems will be necessary before inferring biological mechanism or clinical applicability.

## Conclusion

In conclusion, our study provides robust genetic evidence supporting causal roles for specific circulating miRNAs in allergic rhinitis. We highlight miR-1908−5p as potential protective factors, and miR-130a-3p as a significant driver of AR risk. These findings not only deepen our understanding of the genetic and epigenetic architecture of AR but also suggest that modulating the levels of these key miRNAs could represent a novel therapeutic strategy for the management of allergic rhinitis. However, given the limitations related to tissue specificity, incomplete colocalization support, possible residual pleiotropy, reliance on summary-level data, restriction to predominantly European ancestry datasets, and lack of direct experimental validation, the present results should be interpreted cautiously.

## ORCID IDs

Huaien Zhou: 0009-0004-3353-6554

Jianjun Zou: 0009-0008-8576-0688

Shidong Chu: 0000-0002-0602-9862

## Funding

This study was supported by the Zhejiang Provincial Traditional Chinese Medicine Science and Technology Program Project (Grant nº 2024ZL694).

## Data availability

The authors declare that all data are available in repository.

## Declaration of competing interest

The authors declare no conflicts of interest.

## Appendix A Supplementary data

Table S1 Causal inference analysis results between Rheumatoid Arthritis (RA) and plasma miRNAs based on GCST90476021 data.

Table S2 Colocalization analysis of causal miRNAs and Rheumatoid Arthritis (RA).

Table S3 Reverse Mendelian randomization analysis results of causal miRNAs and Rheumatoid Arthritis (RA).

Table S4 Validation of causal relationships between Rheumatoid Arthritis (RA) and plasma miRNAs based on FinnGen data.

Table S5 Target identification of causal miRNAs validated across multiple Rheumatoid Arthritis (RA) cohorts.
